# Human lymph-node CD8^+^ T cells display an altered phenotype during systemic autoimmunity

**DOI:** 10.1038/cti.2016.8

**Published:** 2016-04-01

**Authors:** Tamara H Ramwadhdoebe, Janine Hähnlein, Bo J van Kuijk, Ivy Y Choi, Leonard J van Boven, Danielle M Gerlag, Paul P Tak, Lisa G van Baarsen

**Affiliations:** 1 Department of Clinical Immunology and Rheumatology, Amsterdam Rheumatology and Immunology Center (ARC), Academic Medical Center/University of Amsterdam, Amsterdam, The Netherlands; 2 Department of Experimental Immunology, Academic Medical Center/University of Amsterdam, Amsterdam, The Netherlands; 3 Department of Radiology, Academic Medical Center/University of Amsterdam, Amsterdam, The Netherlands; 4 Clinical Unit Cambridge, GlaxoSmithKline, Cambridge, UK; 5 University of Cambridge, Cambridge, UK; 6 Ghent University, Ghent, Belgium; 7 GlaxoSmithKline, Stevenage, UK

## Abstract

Although many studies are focused on auto-reactive CD4^+^ T cells, the precise role of CD8^+^ T cells in autoimmunity is poorly understood. The objective of this study is to provide more insight into the phenotype and function CD8^+^ T cells during the development of autoimmune disease by studying CD8^+^ T cells in human lymph-node biopsies and peripheral blood obtained during the earliest phases of rheumatoid arthritis (RA). Here, we show that lymphoid pro-inflammatory CD8^+^ T cells exhibit a less-responsive phenotype already during the earliest phases of autoimmunity compared with healthy individuals. We found an increase in CD8^+^ memory T cells in lymphoid tissue during the earliest phases of autoimmunity, even before clinical onset of RA, accompanied by an increased frequency of non-circulating or recently activated (CD69^+^) CD8^+^ T cells in lymphoid tissue and peripheral blood. Importantly, lymphoid pro-inflammatory CD8^+^IL-17A^+^ T cells displayed a decreased capacity of cytokine production, which was related to disease activity in early RA patients. In addition, a decreased frequency of regulatory CD8^+^IL-10^+^ T cells in peripheral blood was also related to disease activity in early RA patients. Our results suggest that different CD8^+^ T-cell subsets are affected already during the earliest phases of systemic autoimmunity.

Upon antigen recognition naive CD8^+^ T cells differentiate into effector CD8^+^ T cells which are capable of clearing the antigen. After antigen clearance, 90–95% of the effector CD8^+^ T cells undergo apoptosis and only 5% remain present as memory T cells in a quiescent state. These memory CD8^+^ T cells can re-encounter antigen and undergo differentiation into effector memory CD8^+^ T cells. CD8^+^ T cells can also differentiate into a more regulatory phenotype, suppressing CD8 effector functions and thereby dampen the immune response.^1^

Similar to chronic infection and persistent inflammation, (self-) antigens are constantly present. In healthy individuals, T cells are exposed to self-antigens to maintain homeostatic proliferation.^2^ In autoimmune diseases, chronic stimulation of self-reactive CD8^+^ T cells can potentially drive effector CD8^+^ T cells to differentiate into an exhausted phenotype^3^ characterized by low proliferative capacity and low capacity of cytokine production.^4, 5^ Overall, these exhausted T cells display a functional hyporesponsiveness,^4^ which has been observed in several autoimmune diseases including rheumatoid arthritis (RA).^6, 7^

The exact role of CD8^+^ T cells in autoimmune disease is poorly understood. Several studies have shown a contribution of CD8^+^ T cells in autoimmune disease through three different mechanisms.^8^ First, CD8^+^ effector T cells can contribute to autoimmune disease by increased production of pro-inflammatory cytokines^9, 10, 11, 12^ or a decrease in regulatory cytokines.^13^ CD8^+^ T cells can produce IL-17 (Tc17 cells) and lack production of cytolytic granules like granzyme B and perforin.^14^ Therefore, the contribution of Tc17 cells to disease progression is mainly through pro-inflammatory cytokine production. In contrast to Tc17 cells, Tc1 (IFN-γ^+^) and Tc2 (IL-4^+^) cells not only produce cytokines but they are also cytolytic^15^ and can produce granzymes and perforin.

Second, cytotoxic effector CD8^+^ T cells (CTL) can contribute to autoimmune disease by increased tissue infiltration, elevated release of lytic proteins or altered death receptor expression (Fas/CD95) leading to tissue damage.^8^ Besides identifying recently activated T cells, CD69 has been described to distinguish tissue-resident T cells from circulating T cells.^16^ Therefore, CD69 can also be used to study the possible retention of non-circulating CD8^+^ T cells in peripheral tissues and sites of inflammation. Tissue infiltration of CD8^+^ T cells has been described in many autoimmune diseases like type I diabetes (pancreas),^17^ multiple sclerosis (cortical lesions)^18^ and RA (synovial tissue).^19, 20^ In RA, the production of cytolytic proteins by CD8^+^ T cells has been implicated to be critical for ectopic germinal centre formation in synovial tissue.^21^

Third, a dysregulation or decrease in regulatory CD8^+^ T cells can contribute to autoimmune disease.^8^ Different subsets of CD8^+^ T cells that contribute to immune regulation have been described. CD8^+^CXCR3^+^ T cells contribute to immune regulation through IL-10 production.^13, 22^ CD8^+^CD28^−^ T cells can produce IL-10 and TGF-β,^23^ are antigen specific and condition antigen-presenting cells (APC) to become tolerogenic.^24^ On the other hand, the CD8^+^CD28^−^ T cells can express killer immunoglobulin receptors and contain cytotoxic granules and IFN-γ, providing the tools for antigen-independent activation.^2, 25^

As a result of prolonged antigen exposure and increased antigen dose, the role of CD8^+^ T cells during the development of autoimmune disease may change over time. This may result in a shift in balance between regulatory and pro-inflammatory effector CD8^+^ T cells during different phases of the disease and result in an exhausted phenotype of both pro-inflammatory and regulatory CD8^+^ T cells during established disease. Since the development of RA can be preceded by a preclinical phase of systemic autoimmunity,^26, 27, 28^ we can use individuals characterized by anti-citrullinated protein antibodies (ACPA) or IgM rheumatoid factor (IgM-RF) as a model to study the contribution of CD8^+^ T cells in the earliest phases of autoimmune disease.^29^ Such individuals have recently been defined as being RA-risk individuals as recommended by the SGRFRA (Study Group for Risk Factors for RA) under the auspices of the EULAR (the European League Against Rheumatism) Standing Committee of Investigative Rheumatology (ESCIR).^29^ It is expected that 30% of these individuals will develop arthritis within a year.^30^ Furthermore, as the initiation of adaptive immune responses occurs in peripheral lymphoid organs, we aimed to investigate the phenotype and function of CD8^+^ T cells in lymph-node biopsies and peripheral blood obtained during the preclinical RA-risk phase and earliest phases of clinically manifest RA. Previously, we showed that CD8^+^CD69^+^ T cells are increased in lymphoid tissue of RA patients compared with healthy individuals.^31^ More insight into the phenotype and function of CD8^+^ T cells during of the earliest (preclinical) phases of RA will aid to our understanding of their contribution and possible mechanism of action in autoimmunity.

## RESULTS

### Lymphoid cells in the earliest phases of RA contain a higher frequency of memory and non-circulating CD8^+^CD45RA^+^ T cells compared with healthy controls

**Figure 1:**
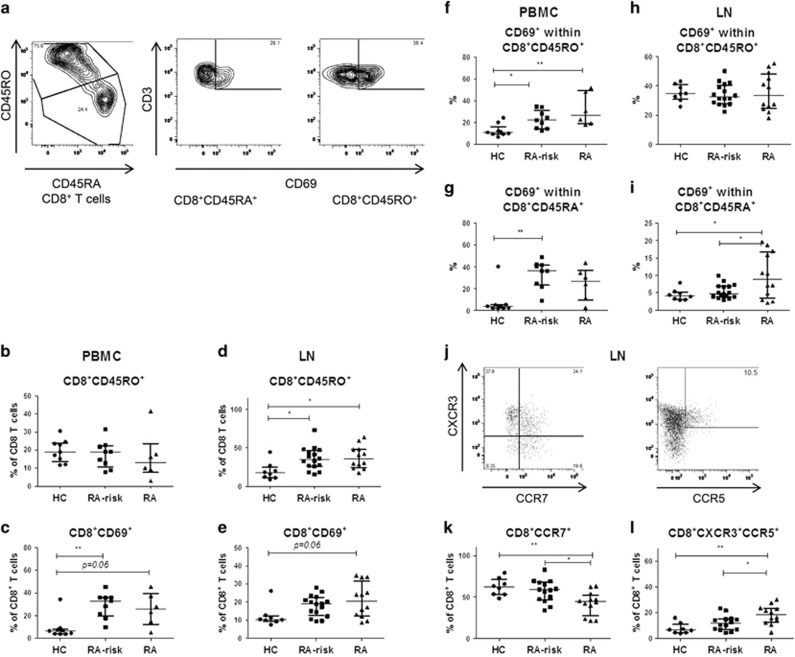
CD8^+^ T-cell phenotype in peripheral blood and lymphoid tissue. Representative flow-cytometry dot plots for gated CD8^+^ memory and tissue-resident T cells from one donor are depicted in (**a**). PBMC (**b**+**c**) and lymphoid memory CD8^+^ T cells (lymph node; **d**+**e**) are analysed based on CD45RO expression and tissue-resident CD8^+^ T cells based on CD69 expression. Data are plotted as frequency of the total CD8^+^ T-cell population. Frequency of CD69^+^ T cells is analysed within both the CD8^+^CD45RO^+^ and CD8^+^CD45RA^+^ populations in PBMC (**f**+**g**) and lymph node (**h**+**i**). PBMC; HC (*n*=9), RA-risk (*n*=9), RA (*n*=6). Lymph node; HC (*n*=8), RA-risk (*n*=16), RA (*n*=12). Representative flow-cytometry dot plots for CD8^+^CCR7^+^ and CD8^+^CXCR3^+^CCR5^+^ T cells are depicted in (**j**). Expression of chemokine receptors for homing (CCR7^+^) and effector phenotype (CXCR3^+^CCR5^+^) is analysed as frequency of positive cells within the CD8^+^ T-cell population in lymph node tissue (**k**+**l**). Lymph node; HC (*n*=8), RA-risk (*n*=14), RA (*n*=12). All data are presented as median with IQR (*P<0.05; **P<0.01).

Because CD45RA^+^ T cells consist of both naive T cells (CD28^+^) and late effector T cells (CD28^+/−^) and CD45RO^+^ T cells are composed of both early differentiated memory (CD28^+^) and late differentiated memory (CD28^+/−^) T cells, we examined the expression of CD28 to differentiate between these subsets.^25^ On the basis of the level of CD28 expression (Supplementary Figure 1) we found that, as expected,^32^ most lymphoid tissue CD8^+^ T cells express high levels of CD28. Thus, CD8^+^CD45RA^+^ T cells in lymphoid tissue consisted mainly of naive T cells and CD8^+^CD45RO^+^ T cells represent mainly early differentiated memory and antigen experienced T cells. In peripheral blood, the CD8^+^CD45RA^+^ cells expressed both high and low levels of CD28, suggesting that CD8^+^CD45RA^+^ population in blood is a combination of late effector and naive CD8^+^ T cells. In the CD8^+^CD45RO^+^ population also both high and low expression of CD28 was detected indicating that the CD8^+^CD45RO^+^ population in peripheral blood consists of both early differentiated and late differentiated memory CD8^+^ T cells. Next, we analysed the expression of CD45RO and CD69 on CD8^+^ T cells in peripheral blood and lymphoid tissue (Figure 1a). In peripheral blood, the frequency of CD8^+^CD45RO^+^ T cells was on average comparable between the different study groups (Figure 1b). The frequency of CD8^+^CD69^+^ T cells was increased in peripheral blood of RA-risk individuals compared with healthy controls (HCs) (*P*=0.006); and a non-significant increase was observed in early RA patients compared with HCs (*P*=0.06) (Figure 1c). In contrast, in lymphoid tissue the frequency of CD8^+^CD45RO^+^ T cells was increased in both RA-risk individuals (*P*=0.03) and early RA patients (*P*=0.02) compared with HCs (Figure 1d). We observed a non-significant increase in the frequency of CD8^+^CD69^+^ T cells in lymphoid tissue of RA patients compared with HC (*P*=0.06) (Figure 1e), which is in line with our previous findings.^31^ In peripheral blood, we found an increase in CD8^+^CD45RO^+^CD69^+^ T cells in RA-risk individuals (*P*=0.04) and early RA patients (*P*=0.01) compared with HCs and an increase in CD8^+^CD45RA^+^CD69^+^ T cells in RA-risk individuals (*P*=0.003) compared with HCs (Figures 1f and g). In lymphoid tissue, there was a significant increase in CD8^+^CD45RA^+^CD69^+^ T cells in early RA patients (*P*=0.02) compared with HCs and compared with RA-risk individuals (*P*=0.02) (Figure 1i). In contrast to CD4^+^ T cells, naïve or memory CD8 subsets do not increase with age in peripheral blood or lymph node.^16^ Accordingly, we did not found any correlation between CD8^+^CD45RA^+^ or CD8^+^CD45RO^+^ T cells and age. Taken together, these data show an increase in CD8^+^ memory and antigen experienced T cells in lymphoid tissue during the earliest phases of RA, even before clinical onset of the disease, which is accompanied by an increased frequency of non-circulating or recently activated (CD69^+^) CD8^+^CD45RA^+^ T cells in lymphoid tissue and increased frequency of (CD69^+^) CD8^+^CD45RO^+^ T cells in peripheral blood.

To investigate homing capacity and effector phenotype of lymphoid CD8^+^ T cells, we analysed the frequency of CD8^+^CCR7^+^ T cells and CD8^+^CXCR3^+^CCR5^+^ effector T cells (Figure 1j). On the basis of chemokine receptor expression, in lymphoid tissue, the frequency of CD8^+^CCR7^+^ cells was decreased in early RA patients compared with HCs (*P*=0.008) and compared with RA-risk individuals (*P*=0.02) (Figure 1k). On the other hand, the frequency of effector CD8^+^CXCR3^+^CCR5^+^ T cells was increased in early RA patients compared with HCs (*P*=0.002) and compared with RA-risk individuals (*P*=0.04) (Figure 1l). Taken together, these data indicate that in early RA patients a decrease in CD8^+^ T cell positive for the lymphoid retention marker CCR7 is accompanied by an increased frequency of CD8^+^ T cell with an effector phenotype based on chemokine receptor expression.

### During the earliest phases of RA, lymphoid CD8^+^ T cells are less capable of producing pro-inflammatory cytokines

**Figure 2:**
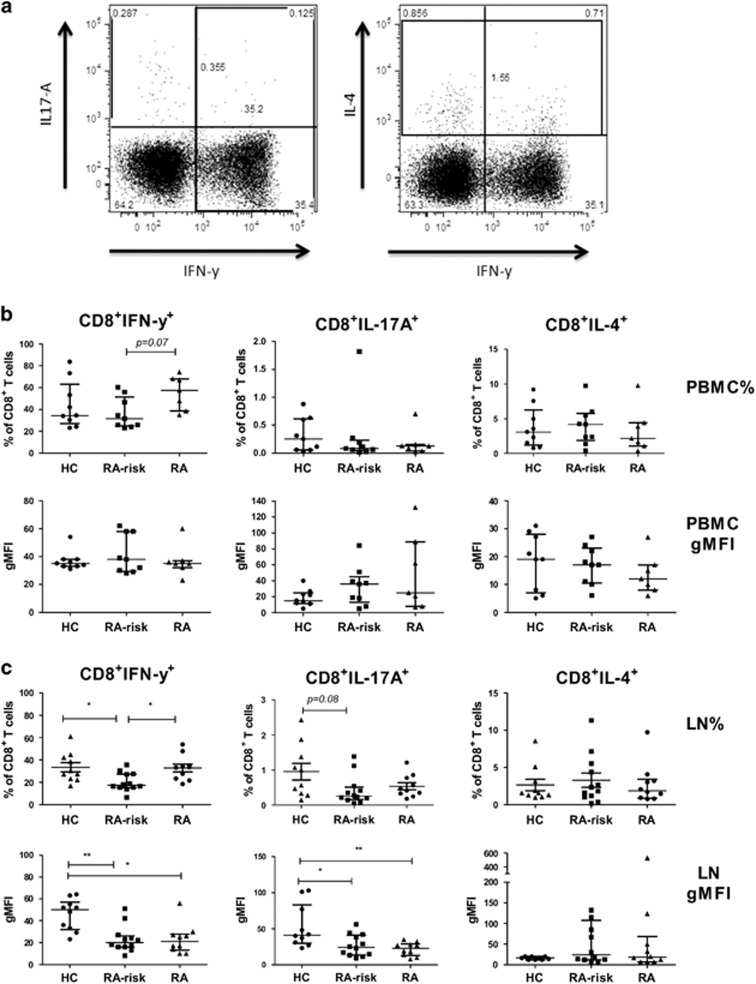
Cytokine production in CD8^+^ T cells in PBMC and lymph node cells. Representative flow-cytometry dot plots for gated CD8^+^ cytokine producing T cells are depicted in (**a**). PBMC and lymph node cells are incubated for 4 h with phorbol myristate acetate (PMA) and Ionomycin in the presence of Brefeldin A and Golgi Stop and stained intracellularly. Frequencies (upper panels) of CD8^+^ T cells producing IFN-γ, IL-17A or IL-4 are analysed using flow cytometry for both PBMC (**b**) and lymph node tissue (**c**). Cytokine production per cell is analysed using gMFI (lower panel) for PBMC and lymph node cells. PBMC; HC (*n*=9), RA-risk (*n*=9), RA (*n*=7). Lymph node; HC (*n*=10), RA-risk (*n*=12), RA (*n*=10). All data are presented as median with IQR (*P<0.05; **P<0.01).

Next, we investigated the frequencies of cytokine producing CD8^+^ T cells and the level of cytokine production in both peripheral blood and lymphoid tissue (Figure 2a). Although cytokine levels are very low in unstimulated cells we first analysed the basal frequencies in both peripheral blood and lymphoid cells (Supplementary Figure 2). The frequency of CD8^+^IFN-γ^+^, CD8^+^IL-17A^+^ and CD8^+^IL-4^+^ T cells in peripheral blood was on average comparable between the different study groups. After stimulation, the frequency of CD8^+^IFN-γ^+^, CD8^+^IL-17A^+^ and CD8^+^IL-4^+^ T cells in peripheral blood was on average comparable between the different study groups (Figure 2b). In addition, no differences were observed for the levels of cytokines produced per peripheral blood cell as determined by the geometric mean fluorescence intensity (gMFI). In contrast, in lymphoid tissue of RA-risk individuals we found a decreased frequency of CD8^+^IFN-γ^+^ T cells compared with HCs (*P*=0.03) (Figure 2c). In early RA patients, this frequency was comparable to HCs. The frequencies of CD8^+^IL-17A^+^ and CD8^+^IL-4^+^ T cells in lymphoid tissue were comparable between the different study groups. The IFN-γ production per CD8^+^ T cell (gMFI) was significantly decreased in RA-risk individuals compared with HCs (*P*=0.004) and in early RA patients compared with HCs (*P*=0.01). In addition, CD8^+^ T cells of RA-risk individuals (*P*=0.01) and early RA (*P*=0.004) patients produced significantly lower levels of IL-17A compared with HCs (Figure 2c).

Taken together, these data indicate that during the earliest phases of RA, lymphoid tissue CD8^+^ T cells produce lower levels of pro-inflammatory cytokines upon *ex vivo* stimulation.

### Frequencies of peripheral and lymphoid cytotoxic effector CD8^+^ T cells are unaltered during the earliest phases of RA

**Figure 3:**
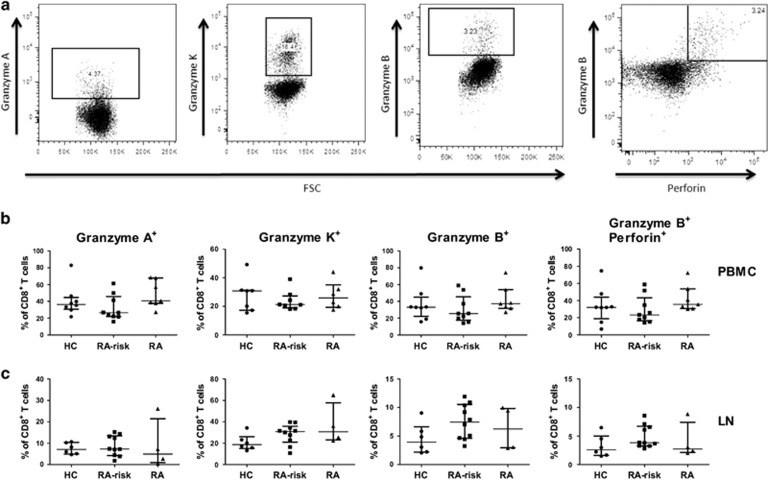
Cytotoxic CD8^+^ T-cell analysis in PBMC and lymph node cells. Representative flow-cytometry dot plots for gated CD8^+^ T cells producing granzyme A, granzyme K, granzyme B and perforin are depicted in (**a**). Frequencies of CD8^+^ T cells producing granzyme A, granzyme K, granzyme B and granzyme B+perforin are analysed using intracellular staining on PBMC (**b**) and lymph node cells (**c**). PBMC; HC (*n*=8), RA-risk (*n*=9), RA (*n*=7) (Granzyme-K HC (*n*=7), RA-risk (*n*=8), RA (*n*=6)). Lymph node; HC (*n*=6), RA-risk (*n*=10), RA (*n*=4). Data are presented as median with IQR.

To study the possible changes in cytotoxic effector CD8^+^ T cells, we investigated the frequency of CD8^+^ T cells positive for cytolytic proteins (Figure 3a). Granzyme A, B and perforin are related to cytolytic activity of CD8^+^ T cells in CTL, while granzyme K is expressed at higher levels in memory CD8^+^ T cells and therefore less related to CTL-mediated toxicity.^33^ We did not find differences in the frequencies of CD8^+^granzyme A^+^, CD8^+^granzyme K^+^, CD8^+^granzyme B^+^ or CD8^+^granzyme B^+^perforin^+^ T cells in peripheral blood (Figure 3b) and lymphoid tissue (Figure 3c) between HCs, RA-risk individuals and early RA patients.

### A decrease in circulating CD8^+^IL-10^+^ is observed during the earliest phases of RA

**Figure 4:**
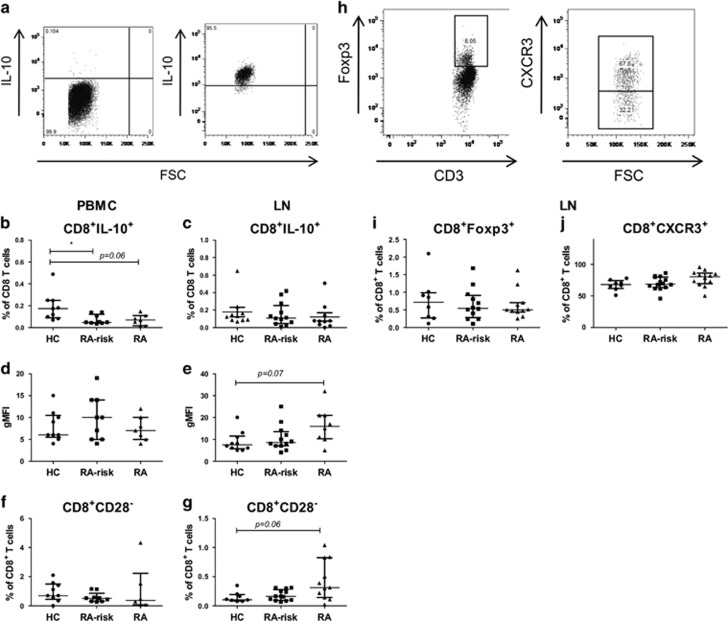
Analysis of different regulatory CD8^+^ T-cell subsets in peripheral blood and lymphoid tissue. Representative flow-cytometry dot plots for gated CD8^+^IL-10^+^ T cells and CD8^+^CD28^−^ T cells are depicted in (**a**). Frequencies (**b**+**c**) and gMFI (**d**+**e**) of CD8^+^IL-10^+^ T cells are analysed in PBMC (left) and lymph node cells (right) after 4 h of stimulation with phorbol myristate acetate (PMA)/Ionomycin in the presence of Brefeldin A and Golgi Stop. Frequencies of CD8^+^CD28^−^ T cells are analysed on unstimulated PBMC (**f**) and lymph node cells (**g**). PBMC; HC (*n*=9), RA-risk (*n*=9), RA (*n*=6 or 7). Lymph node; HC (*n*=8–10), RA-risk (*n*=12–14), RA (*n*=9–12). Representative flow-cytometry dot plots for gated CD8^+^Foxp3^+^ and CD8^+^CXCR3^+^ T cells are depicted in (**h**). Unstimulated lymph node cells are analysed for the frequencies of CD8^+^Foxp3^+^ (**i**) and CD8^+^CXCR3^+^ (**j**) T cells. Lymph node; HC (*n*=8), RA-risk (*n*=12 or 14), RA (*n*=11 or 12). All data are presented as median with IQR (*P<0.05; **P<0.01).

To study the possible changes in regulatory CD8^+^ T cells during the earliest phases of RA, we analysed the presence of different regulatory markers on CD8^+^ T cells (Figure 4a). Although cytokine levels were very low in unstimulated cells, we first analysed the basal frequency of CD8^+^IL-10^+^ T cells in both peripheral blood and lymphoid cells (Supplementary Figure 3). In peripheral blood, we found a significantly lower frequency of CD8^+^IL-10^+^ T cells in early RA patients (*P*=0.04) compared with HCs while frequencies in lymphoid tissue were on average comparable between the different study groups. After stimulation, we found a decreased frequency of CD8^+^IL-10^+^ T cells in peripheral blood of RA-risk individuals compared with HCs (*P*=0.03) and a non-significant decrease for early RA patients compared with HCs (*P*=0.06) (Figure 4b). On the basis of gMFI, the levels of IL-10 produced per CD8^+^ T cell in peripheral blood were on average comparable (Figure 4d). In lymphoid tissue, the frequencies of CD8^+^IL-10^+^ cells were not different between the different study groups (Figure 4c). CD8^+^CD28^−^ T cells can contribute to regulation through IL-10 production. In peripheral blood, we did not find differences in their frequencies between the different study groups (Figure 4f). In lymphoid tissue, we found a non-significant increase in CD8^+^CD28^−^ T cells in early RA patients compared with HCs (*P*=0.06) (Figure 4g). Finally, we analysed the frequency of lymphoid regulatory T cells based on Foxp3 and CXCR3 expression (Figure 4h). The frequencies of both CD8^+^Foxp3^+^ cells (Figure 4i) and CD8^+^CXCR3^+^ cells (Figure 4j) were on average comparable between the different study groups.

Taken together, our data indicate that during the earliest phases of RA the frequency of circulating regulatory CD8^+^IL-10^+^ T cells is decreased.

### Relationship between CD8^+^ T-cell subsets and disease activity in RA

**Figure 5:**
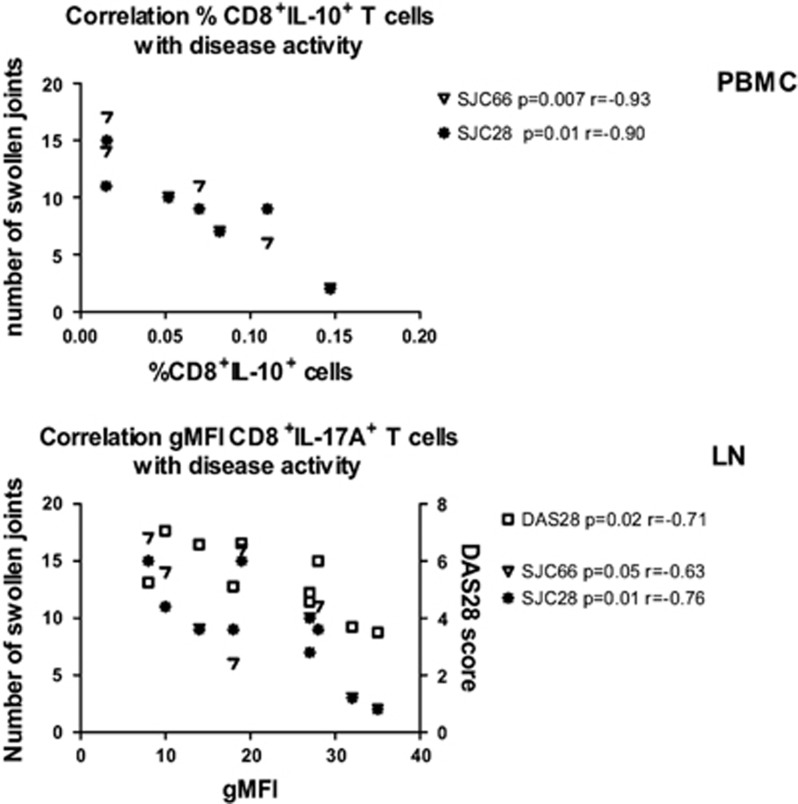
Relationship between CD8^+^ T-cell subsets and disease activity in RA. The correlations between disease activity parameters (SJC66 and SJC28) and the frequency of CD8^+^IL-10^+^ T cells (*n*=7) in PBMC from RA patients are plotted in the upper panel. The correlations between disease activity parameters (DAS28, SJC66, SJC28) and IL-17 production in CD8^+^ T cells measured by gMFI (*n*=10) in lymph node tissue biopsies from RA patients are plotted in the lower panel.

In this exploratory study, we investigated phenotype and function of several CD8^+^ T-cell subsets in lymphoid tissue and peripheral blood of early RA patients. Although this study was not designed to identify the relationship between CD8^+^ T-cell subsets and clinical parameters, we explored their correlation in early RA patients (Figure 5). In peripheral blood, we found a strong and statistically significant negative correlation between the frequency of CD8^+^IL-10^+^ T cells and disease activity parameters, including swollen joint count (SJC) 28 (*P*=0.01, *r*=−0.90) and SJC66 (*P*=0.007, *r*=−0.93). In lymphoid tissue, we found a strong negative correlation between the CD8^+^IL-17A^+^ gMFI and DAS28 (*P*=0.02, *r*=−0.71), SJC66 (*P*=0.05, *r*=−0.63) and SJC28 (*P*=0.01, *r*=−0.76). In addition, there was a negative correlation between the CD8^+^IL-17A^+^gMFI and age in the early RA patients (*P*=0.03, *r*=−0.63; data not shown). These correlations were not present in the RA-risk individuals or HCs. Taken together, these results indicate that during the earliest phases of RA the decreased frequency of circulating regulatory CD8^+^ T cells and the decreased production of pro-inflammatory cytokines upon stimulation of lymphoid CD8^+^ T cells are related to disease activity.

## DISCUSSION

The results presented here show that during the earliest phases of RA, even before the development of clinical signs and symptoms of arthritis, there is an increase in memory and antigen experienced CD8^+^ T cells in lymphoid tissue. In contrast to CD4^+^ memory T cells, CD8^+^ memory T cells do not accumulate in lymph nodes or blood during ageing.^16^ Therefore, it is plausible that this increase in lymphoid CD8^+^ memory T cells is disease related rather than age related. We postulate that as a result of continuous antigen presentation and consequent stimulation *in vivo*, pro-inflammatory lymphoid CD8^+^ T cells display a less-responsive phenotype based on their decreased capacity to produce cytokines *ex vivo*. During the RA-risk phase and early RA phase, we observed a decrease in IFN-γ and IL-17A production in lymphoid CD8^+^ T cells. In addition, we observed a decreased frequency of circulating regulatory CD8^+^IL-10^+^ T cells in the RA-risk and early RA phase. In early RA patients, the decreased production of IL-17A by lymphoid CD8^+^ T cells and the decreased frequency of circulating CD8^+^IL-10^+^ T cells were associated with increased disease activity. In addition, there was also a clear correlation with disease activity scores. Because of the challenging procedure of obtaining lymph-node biopsies from healthy individuals, RA-risk individuals and RA patients, the number of analysed individuals is relatively small. Future studies are needed to validate our findings.

T-cell activation is accompanied by downregulation of CCR7 in lymphoid effector T cells to facilitate lymph-node egress.^34^ We found a decreased frequency of CD8^+^CCR7^+^ T cells in lymphoid tissue of early RA patients, which may reflect a shift towards more migratory effector CD8^+^ T cells leaving the lymph node to travel towards sites of inflammation. We found an increase in CD8^+^ T-cell subtypes in both lymphoid tissue and peripheral blood positive for CD69 (potentially tissue-resident T cells^16^). Although CD69 is known to be an early activation marker, expression of CD69 on CD8^+^ T cells in lymphoid tissue may indicate retention inside the lymph node where T cells may encounter antigen and become activated. In peripheral blood, expression of CD69 on CD8^+^ memory T cells may indicate an increase in activated T cells possibly travelling towards sites of inflammation. In line with our data, previous studies have reported an increase in memory CD8^+^ T cells in peripheral blood^35^ and synovial fluid^36^ of RA patients. Previously, our group has demonstrated that there appears to be a slightly increased infiltration of CD8^+^ T cells in synovial tissues of RA-risk individuals who developed arthritis later on.^37^ These data suggest that CD8^+^ T cells may have homing capacity to the synovium already very early in the disease. The phenotype and function of these synovial CD8^+^ T cells during the preclinical phase needs to be studied in detail to understand whether these CD8^+^ T cells are regulatory or pro-inflammatory and therefore possibly involved in the initiation of the disease.

We studied the capacity of CD8^+^ T cells in producing pro-inflammatory cytokines upon stimulation and observed a decrease in IFN-γ and IL-17A production in lymphoid CD8^+^ T cells already in the RA-risk phase and in early RA patients. This diminished capacity of cytokine production is characteristic for exhausted CD8^+^ T cells, and can be a result of prolonged antigen exposure followed by T-cell hyporesponsiveness.^4^ Further analyses of CD8^+^ T cells, using specific markers for exhaustion, are required to formally test whether these CD8^+^ T cells are indeed exhausted during the earliest phases of RA.

Regulatory CD8^+^IL-10^+^ T cells have been studied in RA because of their possible role in dampening the inflammatory immune responses. The frequency of CD8^+^IL-10^+^ cells is higher in synovial fluid of RA patients compared with peripheral blood, suggesting that these synovial fluid cells attempt to modulate local inflammation through IL-10 production in the inflamed tissue.^36, 38^ The current study showed a decreased frequency of circulating CD8^+^IL-10^+^ T cells in the preclinical RA-risk phase and early RA patients, which may contribute to impaired resolution of inflammation and autonomous disease progression.^39^ It has been reported that granzyme B levels in synovial tissue are increased in RA patients and correlate with severity of joint damage.^40, 41^ The source of this cytotoxic enzyme appears to be natural killer cells rather than cytotoxic T cells in early RA.^42^ Accordingly, we found that the frequencies of effector CD8^+^ T cells positive for cytolytic proteins are unaltered in both peripheral blood and lymphoid tissue during the earliest phases of RA, but we did not examine the production by natural killer cells.

Taken together, our data clearly demonstrate that CD8^+^ T cells are affected during different stages of autoantibody-positive RA. Longer follow-up time of the RA-risk individuals will provide more insight into the possible contribution of the different CD8^+^ T-cell subtypes towards the development of arthritis. The observed changes in both pro-inflammatory and regulatory CD8^+^ T cells already in individuals with evidence of autoimmunity imply an important role for CD8^+^ T cells early in the pathogenesis of autoimmune diseases, which needs further attention.

## Methods

### Study subjects

We included 20 individuals at risk for developing RA^29^ (RA-risk individuals defined by IgM-RF positivity or ACPA positivity). IgM-RF was measured using IgM-RF ELISA (Sanquin, Amsterdam, The Netherlands; ULN (upper limit of normal) 12.5 IU ml^−1^) until December 2009 and thereafter using IgM-RF ELISA (Hycor Biomedical, Indianapolis, IN, USA (ULN 49 IU ml^−1^). ACPA was measured using anti-CCP2 ELISA CCPlus (Eurodiagnostica, Nijmegen, The Netherlands (ULN 25 kAU l^−1^)). Median follow-up time of RA-risk individuals is 19.7 months and none of the RA-risk individuals developed arthritis during this period. We included 17 early RA patients, diagnoses based on the American College of Rheumatology and EULAR (ACR/EULAR) 2010 criteria, naive for disease-modifying antirheumatic drugs and biologicals with a disease duration (defined by arthritis in any joint) less than 1 year. For comparison, 19 seronegative HCs were included in the study. The study was performed according to the principles of the Declaration of Helsinki, approved by the institutional review board of the Academic Medical Centre, and all study subjects gave written informed consent. Demographics of all study subjects are listed in Table 1.

### Sample processing and cell culture

Ultrasound guided inguinal lymph-node biopsies were taken and processed as described earlier.^43^ Briefly, lymph-node biopsies were put through a 70-μm cell strainer (BD Falcon, San Jose, CA, USA) to obtain a single cell suspension. If possible, paired peripheral blood samples were obtained on the same day as lymph-node biopsies were taken. This was not allowed if blood had already been drawn for other purposes on an earlier time point close to study visit date. Peripheral blood mononuclear cells (PBMC) were isolated using standard density gradient centrifugation with lymphoprep (Nycomed AS, Oslo, Norway) and stored in liquid nitrogen until further use. Freshly isolated lymph-node cells or thawed PBMC were incubated in RPMI culture medium (Life Technologies, Thermo Fisher Scientific Inc., Waltham, MA, USA) for 4 h in the presence or absence of Phorbol Myristate Acetate and Ionomycine with Brefeldin A (all from Sigma-Aldrich, St Louis, MO, USA) and Golgi stop (BD Biosciences, San Jose, CA, USA). After 4 h, cells were washed and analysed by flow cytometry.

### Antibodies and flow-cytometry analysis

Cells were stained for 30 min at 4 °C in PBS containing 0.01% NaN_3_ and 0.5% BSA with directly labelled antibodies against: CXCR3 alexa fluor488, CCR5 PE, CCR4 PercP-Cy5.5, CCR7 PE-Cy7, CCR6 alexa647, CD4 APC-H7,CD8 V500, CD3 V500, CD69 PercP, CD45 V500, Foxp3 PercP-Cy5.5, Granzyme-A PE, CD8 V450 (all from BD Biosciences), CD3 FITC (Sanquin, Amsterdam, The Netherlands), CD4 PE-Cy7, CD28 APC, CD8 APC-efluor780, CD45RA efluor450, CD45RO PE (all from eBioscience Inc., San Diego, CA, USA), Granzyme-K Fitc (Immunotools, Friesoythe, Germany), Granzyme-B APC (Invitrogen, Thermo Fisher Scientific Inc.) and Perforin PercP-Cy5.5, IL-10 Pe-Cy7 (Biolegend, San Diego, CA, USA). For cytokine staining, we used the Th1/Th2/Th17 kit from BD Biosciences. Cells were analysed on an FACS Canto II (BD Biosciences) and data were analysed using the FlowJo software (FlowJo, Ashland, OR, USA).

Data were plotted as frequency of positive cells or as the gMFI to illustrate cytokine expression levels. To correct for experimental variation, the gMFI of cells of interest was normalized to the gMFI of the negative population.

### Statistical analysis

Data are presented as median with interquartile range (IQR). Normally distributed data were analysed using one-way analysis of variances (ANOVA) with post-Bonferroni's multiple comparison tests. Not normally distributed data were analysed using a Kruskall–Wallis followed by Dunns multiple comparison test. Correlations were calculated using Spearman's rho. All statistical analyses were performed using the GraphPad Prism Software (version 6, GraphPad Software, Inc., La Jolla, CA, USA).

## Figures and Tables

**Table 1:** Baseline characteristics of healthy controls (HC), RA-risk individuals and early RA patients

	*HC*	*RA-risk*	*Early (RA)*
	*n*=*19*	*n*=*20*	*n*=*17*
Sex, female (%)	14 (74)	18 (90)	10 (59)
Age (years) (median (IQR))	30.0 (26.0–36.3)	50.0 (46.0–57,5)	57.0 (47.0–59.8)
IgM-RF positive (*n* (%))	0 (0)	10 (50)	16 (94)
IgM-RF level (kU l^−1^) (median ((IQR))	7.5 (1.0–15.0)	51.0 (11.5–272.0)	312.0 (159.5–510.5)
ACPA positive (*n* (%))	0 (0)	10 (50)	15 (88)
ACPA level (kAU l^−1^) (median (IQR))	4.0 (2.0–8.3)	13.0 (6.0–79.0)	388.0 (88.5–1735.5)
IgM-RF and ACPA both pos. (*n* (%))	0 (0)	0 (0)	14 (82)
ESR (mm h^−1^) median (IQR))	nd	8.0 (5.0–14.0)	19.5 (5.8–32.0)
CRP (mg l^−1^) (median (IQR))	0.7 (0.4–1.7)	1.9 (0.9–4.3)	7.1 (4.2–13.9)
68 TJC (*n*) (median (IQR))	0 (0)	2.0 (1.0–4.0)	13.0 (3.8–19.3)
66 SJC (*n*) (median (IQR))	0 (0)	0 (0)	7.0 (4.3–10.0)
DAS28 (median (IQR))			4.6 (3.8–5.8)

Abbreviations: ACPA, anti-citrullinated protein antibodies; CRP, C-reactive protein; ESR, erythrocyte sedimentation rate; IgM-RF, IgM rheumatoid factor; IQR, interquartile range; nd, not determined; RA, rheumatoid arthritis; 68 TJC, tender joint count of 68 joints; 66 SJC, swollen joint count of 66 joints.

Categorical variables: *n* (%). Continuous variables (data not normally distributed): median (IQR).

## Notes

PPT and DMG are currently also employee at GlaxoSmithKline, UK. GSK was not involved in this study.
