# Supplementary material for: Human lymph-node CD8+ T cells display an altered phenotype during systemic autoimmunity
Source: Clin Transl Immunology. 2016 Apr 1;5(4):e67–. doi: 10.1038/cti.2016.8 (PMC4855272; doi:10.1038/cti.2016.8)
Supplement: Supplementary Information [file cti20168x1.doc]

**Text summary Supplementary Information**

**Supplemental Figure 1| Expression of CD28 on CD8^+^ T cells in PBMC and LN cells.** CD28 expression is analysed on CD8^+^CD45RA^+^ T cells and the CD8^+^CD45RO^+^ T cells in lymphoid cells (upper panel) and in peripheral blood (lower panel). Circles indicate analysis of CD28 expression on gated populations.

**Supplemental Figure 2|** **Analysis of cytokine production in unstimulated PBMC and LN cells.** The frequencies of CD8^+^IFN-γ^+^, CD8^+^IL-17A^+^ and CD8^+^IL-4^+^ T cells in PBMC (upper panel) and LN cells (lower panel) are plotted as frequency of the total CD8^+^ population. PBMC; HC (n=10), RA-risk (n=12), RA (n=10). LN; HC (n=9), RA-risk (n=9), RA (n=7). Data are presented as median with IQR; (* p<0.05; **p<0.01).

**Supplemental Figure 3|** **Analysis of IL-10 production in unstimulated PBMC and LN cells.** The frequencies of CD8^+^IL-10^+^ T cells in PBMC (upper panel) and LN cells (lower panel) are plotted as frequency of the total CD8^+^ population. PBMC; HC (n=9), RA-risk (n=9), RA (n=7). LN; HC (n=10), RA-risk (n=12), RA (n=10). Data are presented as median with IQR; (* p<0.05; **p<0.01).
