# Supplementary figures and images for: Human lymph-node CD8+ T cells display an altered phenotype during systemic autoimmunity
Source: Clin Transl Immunology. 2016 Apr 1;5(4):e67–. doi: 10.1038/cti.2016.8 (PMC4855272; doi:10.1038/cti.2016.8)

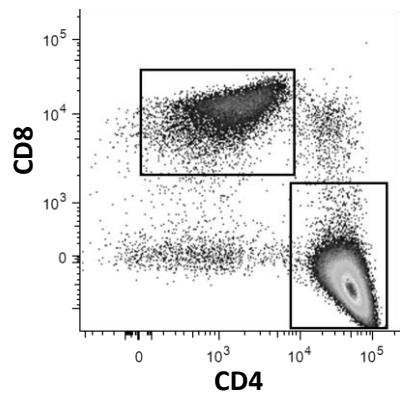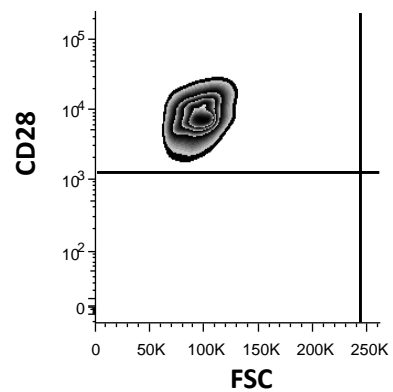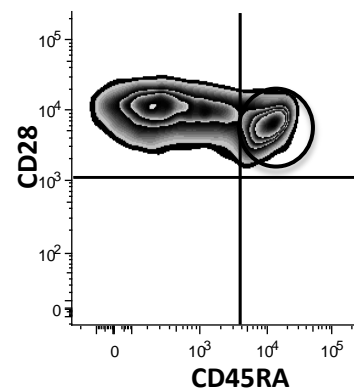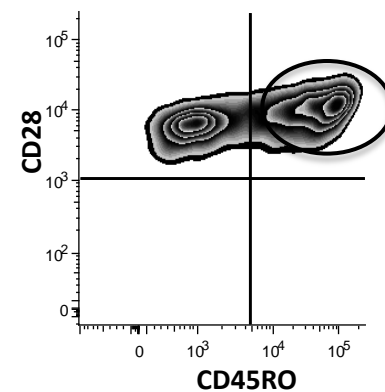

LN

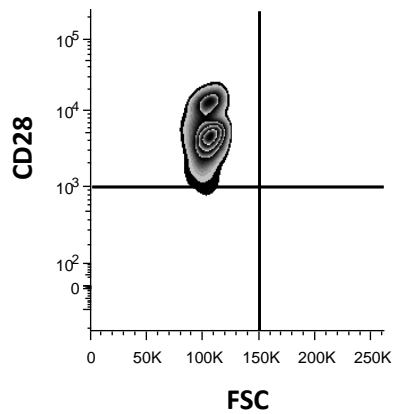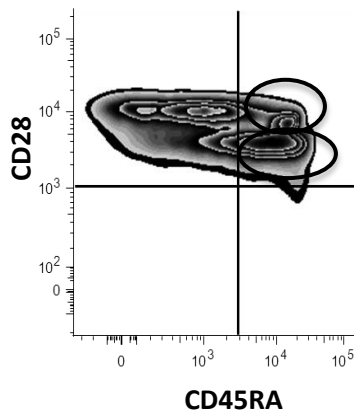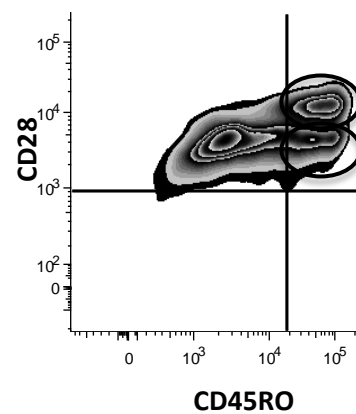

PBMC

Supplement: Supplementary Figure 1 [file cti20168x2.pdf]

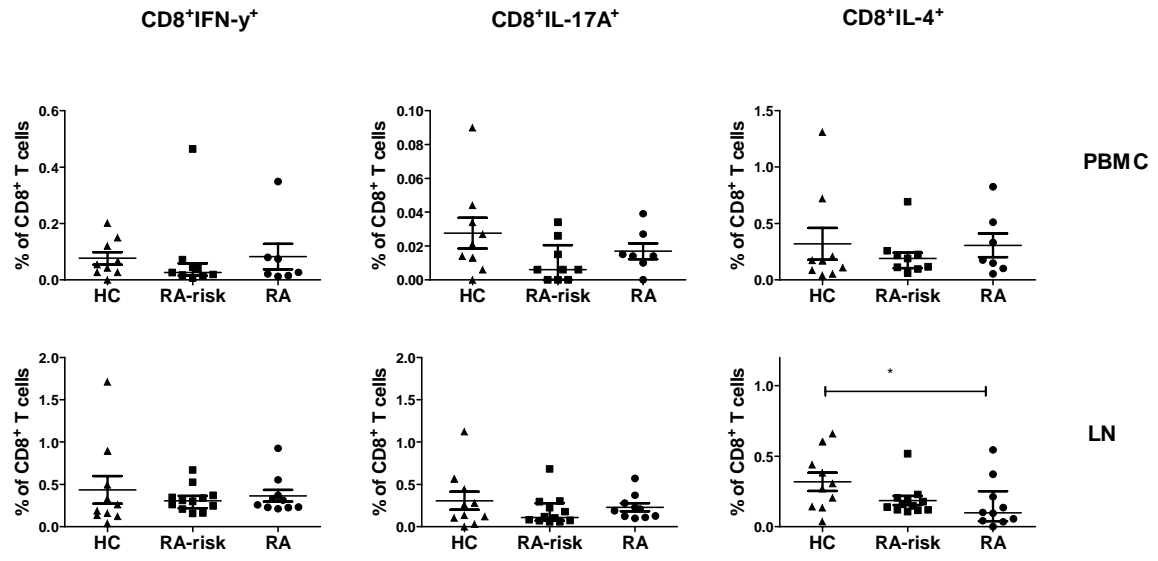

Supplement: Supplementary Figure 2 [file cti20168x3.pdf]

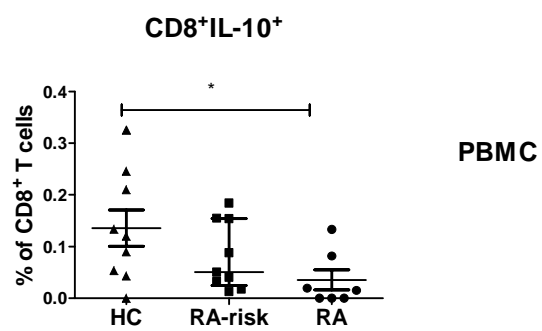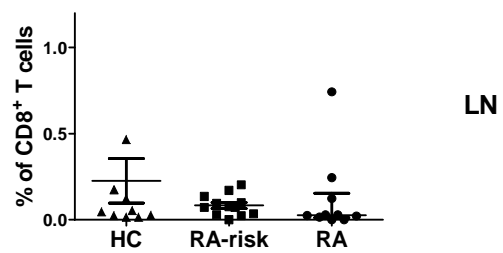

Supplement: Supplementary Figure 3 [file cti20168x4.pdf]
